# Foraging bumblebees acquire a preference for neonicotinoid-treated food with prolonged exposure

**DOI:** 10.1098/rspb.2018.0655

**Published:** 2018-08-29

**Authors:** Andres N. Arce, Ana Ramos Rodrigues, Jiajun Yu, Thomas J. Colgan, Yannick Wurm, Richard J. Gill

**Affiliations:** 1 Department of Life Sciences, Imperial College London, Silwood Park Campus, Buckhurst Road, Ascot SL5 7PY, UK; 2 Department of Organismal Biology, Queen Mary University of London, Mile End Road, London E1 4NS, UK

**Keywords:** aversion, chronic exposure, hazard, insect pollinator, risk, thiamethoxam

## Abstract

Social bees represent an important group of pollinating insects that can be exposed to potentially harmful pesticides when foraging on treated or contaminated flowering plants. To investigate if such exposure is detrimental to bees, many studies have exclusively fed individuals with pesticide-spiked food, informing us about the hazard but not necessarily the risk of exposure. While such studies are important to establish the physiological and behavioural effects on individuals, they do not consider the possibility that the risk of exposure may change over time. For example, many pesticide assays exclude potential behavioural adaptations to novel toxins, such as rejection of harmful compounds by choosing to feed on an uncontaminated food source, thus behaviourally lowering the risk of exposure. In this paper, we conducted an experiment over 10 days in which bumblebees could forage on an array of sucrose feeders containing 0, 2 and 11 parts per billion of the neonicotinoid pesticide thiamethoxam. This more closely mimics pesticide exposure in the wild by allowing foraging bees to (i) experience a field realistic range of pesticide concentrations across a chronic exposure period, (ii) have repeated interactions with the pesticide in their environment, and (iii) retain the social cues associated with foraging by using whole colonies. We found that the proportion of visits to pesticide-laced feeders increased over time, resulting in greater consumption of pesticide-laced sucrose relative to untreated sucrose. After changing the spatial position of each feeder, foragers continued to preferentially visit the pesticide-laced feeders which indicates that workers can detect thiamethoxam and alter their behaviour to continue feeding on it. The increasing preference for consuming the neonicotinoid-treated food therefore increases the risk of exposure for the colony during prolonged pesticide exposure. Our results highlight the need to incorporate attractiveness of pesticides to foraging bees (and potentially other insect pollinators) in addition to simply considering the proportion of pesticide-contaminated floral resources within the foraging landscape.

## 1. Introduction

Pesticides play an important role in ensuring food security by helping to maintain high yielding and healthy agricultural crops. However, the application of these chemicals, in particular insecticides, may inadvertently harm non-target insect species such as insect pollinators that benefit flowering crops and wildflowers [1–3]. Developing solutions to address this concern requires us to study not only how hazardous these chemicals are to insect pollinators, but also to understand the extent to which wild pollinators will be exposed to them [4–7]. Bees are a vitally important group of insect pollinators and are considered to be under threat globally [1,8–11]. In particular, pesticides are thought to contribute to bee population declines because (i) bees forage across landscapes where the application of pesticides are common throughout the year [12], (ii) pesticide residues can be detected in bee-collected pollen, nectar, and within the food stores of social bee colonies (see electronic supplementary material, table S1), and (iii) experimental evidence suggests that exposure to field realistic pesticide concentrations affects bee fitness [13–17].

There has been much contention surrounding the risk of exposure to neonicotinoid insecticides. When directly applied to crops (typically as a seed treatment) or as contamination of wildflowers, this can result in low-level contamination of nectar and pollen at concentrations in the parts per billion (ppb), providing a direct route of exposure to foraging bees [6,18]. An increasing number of studies investigating the effects of neonicotinoid exposure on bees have reported detrimental impacts on a range of behaviours [19–21], including individual foraging performance, which could impair colony development in social bees [22,23]. While laboratory and semi-field studies have helped to elucidate the potential hazards posed by neonicotinoids [20], provisioning bees solely with treated food can potentially result in higher exposure than in the field. For example, many insects can detect a host of chemicals that act as phago-deterrents [24] which can be genetically determined or produced by a learning process [25]. Thus, if bees can detect the presence of pesticides, they may be able to avoid consuming them through either an innate or learned aversion to a novel pesticide in the environment. We are aware of only one study that has tested bumblebee preference for neonicotinoid insecticides [5], which was conducted across a short temporal period, therefore a significant gap remains in our understanding of how foraging bees react to the chronic presence of neonicotinoids in a variable ‘pesticide landscape’ [26].

The introduction of a novel, harmful, compound in an environment where uncontaminated food is available can lead to three primary outcomes in terms of bee foraging behaviour: (i) no change in behaviour, resulting in the harmful compound being consumed in proportion to its prevalence in the environment (*maladaptive*); (ii) a rejection of contaminated food, whereby foragers avoid the harmful compound and therefore reduce their exposure (*adaptive*); or (iii) an increased consumption of the contaminated food whereby bees develop an acquired preference, thus increasing exposure to the harmful compound (*maladaptive*).

The ability of an insect to react to a pesticide, however, depends on its ability to detect the presence of the chemical [27]. A recent electrophysiological study [5] showed that honeybees and bumblebees are unable to taste three major neonicotinoids (clothianidin, imidacloprid and thiamethoxam) through their proboscis. However, the same study showed that isolated bees consumed a higher total amount of pesticide-laced sucrose solution containing either imidacloprid or thiamethoxam than untreated sucrose solution in a two-choice feeding assay over 24 h. This result could be explained by an innate preference of individual bees towards neonicotinoid-contaminated food. However, as to how this behaviour in isolated bees relates to the foraging dynamics of a whole bee colony is unclear. For example, foraging bees can change their behaviour as they gain experience, allowing them to adapt to new information about their environment [28]. This ability to respond to environmental cues can even reverse innate preferences. For instance, bumblebees can rapidly overcome their innate preference towards the colour blue and become attracted to other colours if they are associated with a reward [29] allowing the potential for behavioural avoidance of chemicals that can cause harm.

Single or short-term acute exposure is likely to be rare in the wild. Neonicotinoids can persist in the environment for extended periods of time within the pollen and nectar of treated crops, or as soil residues which degrade slowly (half-life: 148–6900 days; [30]) and can contaminate nearby non-treated wildflowers [7,31]. Also, even across small geographical distances, neonicotinoid concentrations can be heterogeneous, with concentrations in nectar and pollen varying by as much as 4.5 times between a treated crop and adjacent wildflowers [7]. The combination of low but variable amounts of neonicotinoids in the environment means that at field realistic concentrations, the detrimental effects can take an extended period of time to become apparent [23,32]. Indeed understanding the effects of chronic exposure on bee behaviour has been identified as a priority by the European Food Standards Agency [33]. In sum, empirical testing of how food contaminated with a range of neonicotinoid concentrations affects the dynamics of foraging behaviour over a prolonged period of time is required.

**Figure 1.**
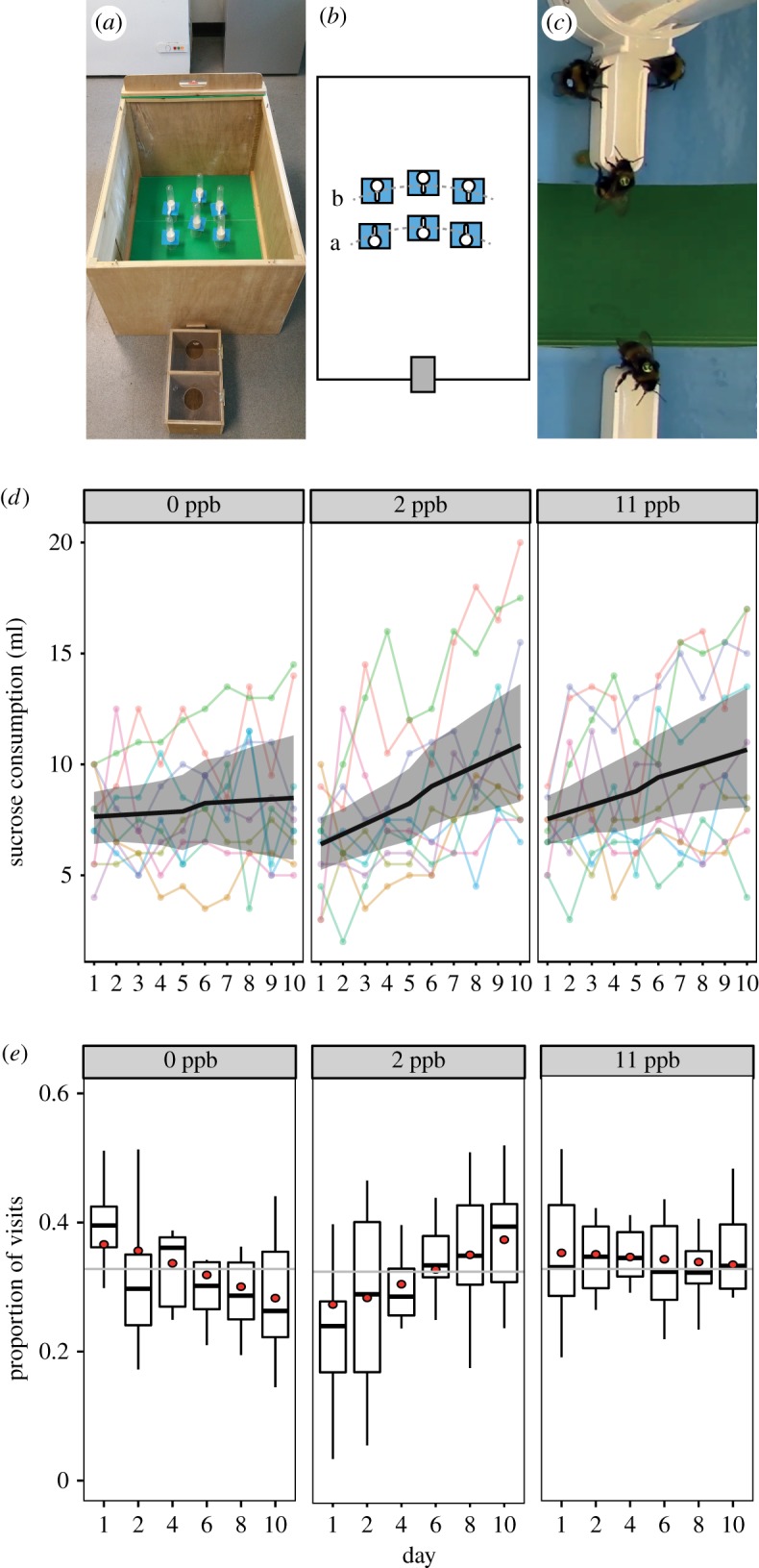
(*a*) Experimental set-up showing the wooden nest box attached to a foraging arena; (*b*) each colony was provided with a choice of six feeders (two per concentration) placed in two rows positioned (*a*) 50 and (*b*) 60 cm from the entrance to the arena (grey rectangle); (*c*) video image showing tagged bees feeding. We also present colony level data for (*d*) the volume of sucrose consumed from each concentration (*n* = 300) and (*e*) boxplots showing the median and interquartile range for the proportion of observed foraging visits to each concentration (*n* = 9542). Circles represent the back transformed mean predictions from the mixed effects models and the grey line represents the null expectation for bees visiting each concentration equally. (Online version in colour.)

We conducted an experiment that provided foraging bumblebees access to sucrose containing 0, 2 and 11 ppb of thiamethoxam (figure 1*a–c*) in a three-choice assay. For this, we used 10 queenright bumblebee colonies attached to a foraging arena over 10 days. For each colony, we measured the volume of sucrose consumed and the proportion of visits to each concentration. Furthermore, we tracked the behaviour of a subset of individually tagged workers. Here, we present data on the dynamics of foraging behaviour on three concentrations of thiamethoxam over time to test whether foragers adapt their behaviour to reduce their exposure to this harmful pesticide. We further test the ability of bumblebees to detect and behaviourally respond to changes in the foraging environment by changing the spatial arrangement of each concentration midway through the experiment.

## 2. Methods

### (a) Experimental set-up and measuring sucrose consumption

Ten *Bombus terrestris audax* (Agralan, UK) colonies (electronic supplementary material, table S2) were each connected to separate flight arenas (L100 × W70 × H50 cm; figure 1*a*), and allowed to forage for 6 h per day between 10.00 and 16.00. When the colonies arrived, we tagged every bee with a unique identification number (*n* = 381), allowing us to identify individuals that had access to the thiamethoxam for the entire duration of the experiment. Each arena contained six gravity feeders each containing 20 ml of a 30% (w/v) sucrose solution positioned in two rows of three at 50 and 60 cm from the entrance (figure 1*b*). After an initial training phase (electronic supplementary material, figure S1), one feeder in each row of feeders was randomly assigned to each of the three concentrations. The concentration assigned to each location remained unchanged for the first five days (Period 1), and on the sixth day we re-randomized the spatial positions (which could not be in the same order as in Period 1) and these remained in place until the end of the experiment (the end of Period 2). Each day, immediately before bees were allowed to access the arena, we placed 2 g of honeybee collected pollen directly into the colony and placed feeders containing fresh sucrose solutions from each concentration into their allocated position (total provided per arena = 120 ml per day). At 16.00 we removed the feeders, measured the remaining volume of sucrose (±0.5 ml), and cleaned the feeders first with 70% ethanol and then water to remove any neonicotinoid residues or scent cues.

### (b) Behavioural observations

We video-recorded the foraging behaviour of the bumblebees (HC-V160, Panasonic, Japan; figure 1*c*) in each colony for the first 3 h of foraging (10.00–13.00) on days 1, 2, 4, 6, 8 and 10. We watched the 180 h of video footage and recorded (i) the number of foraging visits, defined as a visit to a feeder where the bee extended its proboscis into the sucrose solution for ≥ 5 s (*n* = 9542); (ii) the duration of any foraging visit, defined as the time between the first extension of the proboscis into the sucrose solution to its retraction (excluding video footage of day 1, *n* = 8622); and (iii) the tag number and number of visits made by any tagged individual.

### (c) Data analysis

Our null expectation is that, in the absence of attractive or aversive properties of any thiamethoxam concentration, bees should visit, and consume sucrose, from each concentration equally. The data were analysed in R v. 3.0.1, using mixed effects models in the lme4 package [34], to determine how colony level (*n* = 10) foraging is influenced by exposure to the pesticide concentration (*treatment*), length of exposure (*day*) and the interaction between the two (*treatment* × *day*). Our analyses accounted for any inherent colony level differences by including *colony* as a random intercept, while fitting a random slope using *day* as a continuous random variable to represent repeated measures. We analysed the daily volume of each concentration consumed during the experiment using a Gaussian distribution.

Our observation data included monitoring every foraging visit during the same 3 h period every day. This allowed us to model the proportion of visits to each concentration using a binomial distribution. We further analysed the proportion of visits that individual tagged bees made to each feeder through the experiment. For this, we limited the dataset to ‘committed foragers’, which we defined as any tagged individual observed foraging on at least three separate days and that contributed to at least 1% (minimum, 47 observations) of the observed foraging visits from tagged individuals (*n* = 31). We included the individual (*tag*) as a random effect nested within *colony* as a random intercept and *day* as a random slope to account for repeated measures within individuals. In all models, where we report percentages or volumes, these are model estimates back transformed from the model predictions, and all model outputs are relative to the 0 ppb which represents our reference group.

## 3. Results

### (a) Sucrose consumption

The colonies initially consumed similar amounts of 0 ppb and 11 ppb thiamethoxam (7.64 ml and 7.53 ml, respectively; LMM: *t* = −0.659, *p* = 0.510, electronic supplementary material, table S3), while consuming significantly less 2 ppb thiamethoxam compared with 0 ppb (6.39 ml, *t* = −3.016, *p* = 0.003). Throughout the experiment, the amount of sucrose consumed per day increased, probably due to colonies increasing in size as new workers eclosed (figure 1*d*). However, the rate of increase varied significantly between concentrations: the consumption of the 0 ppb concentration increased at a rate of just 0.05 ml per day, while the consumption of the 2 ppb and 11 ppb concentrations increased at 0.45 and 0.34 ml per day, respectively. The rates of consumption for both thiamethoxam concentrations were significantly higher than for the 0 ppb sucrose (2 ppb: *t* = 4.559, *p* < 0.001; 11 ppb: *t* = 2.882, *p* = 0.004). By the last (tenth) day of the experiment, bumblebees had consumed 28% and 26% more of each of the thiamethoxam concentrations relative to the 0 ppb (0 ppb = 8.49 ml, 2 ppb = 10.85 ml and 11 ppb = 10.66 ml).

### (b) Foraging visits to concentrations

In line with the consumption data, our observations of foraging visits showed no evidence of an initial preference for thiamethoxam. At the start of the experiment, the proportion of visits to the 0 ppb and 11 ppb concentrations were similar (36.8% and 35.49%, respectively; GLMM: *z* = −1.374, *p* = 0.169; electronic supplementary material, table S4, figure 1*e*; for count data see electronic supplementary material, figure S2 and table S5) and there was a significantly lower proportion of visits to the 2 ppb (27.9%) relative to the 0 ppb concentration (*z* = −7.467, *p* < 0.001). The proportion of visits to the 0 ppb concentration decreased throughout the experiment (−0.831% per day, *z* = −5.80, *p* < 0.001), and in turn there was a corresponding increase in preference for thiamethoxam. Specifically, the coefficients for the effect of *day* on each thiamethoxam concentration were significantly higher than the 0 ppb concentration (2 ppb: *z* = 9.508, *p* < 0.001; 11 ppb: *z* = 3.431, *p* < 0.001) resulting in an increased proportion of visits to the 2 ppb concentration by 1.004% per day, and with little change in the 11 ppb concentration of −0.183% per day. Consequently, by the final day, the proportion of visits by bees to each concentration was 28.49% to 0 ppb, 37.94% to 2 ppb and 33.6% to 11 ppb. We also analysed the duration of 8622 foraging visits and found that, while the durations decreased as the experiment progressed (rate of −1.9 s per day; LMM: *t* = −3.101, *p* = 0.002) there was no difference in feeding time between concentration groups (*t* ≤ 1.508, *p* > 0.05; electronic supplementary material, table S6 and figure S3). This suggests that the consumption rate of foragers was similar across concentrations.

We next repeated our analysis of foraging visits based on tagged individuals that we considered committed foragers. These bees initially visited the 0 ppb (41.99%) concentration more frequently than either of the two pesticide concentrations (2 ppb: 27.03%; *z* = –9.508, *p* < 0.001; 11 ppb: 35.26%, *z* = −5.341, *p* < 0.001; electronic supplementary material, table S7 and figure S4) suggesting an initial aversion to both concentrations of thiamethoxam. As the experiment progressed, the temporal dynamics of foraging behaviour in the tagged individuals mirrored the observations at the whole colony level. The proportion of visits to the 0 ppb concentration declined at a rate of −1.6% per day (*z* = −4.822, *p* < 0.001), and in turn the rates of change in the proportion of visits to both thiamethoxam concentrations were significantly higher than the proportion of visits to the 0 ppb concentration (2 ppb: *z* = 8.735, *p* < 0.001; 11 ppb: *z* = 5.734, *p* < 0.001). The proportion of visits to the 2 ppb concentration increased by 1.16% per day while the proportion of visits to the 11 ppb concentration remained relatively constant, increasing by 0.22% per day. By the end of the experiment the proportion of visits to each concentration was 27.93% to 0 ppb, 37.46% to 2 ppb and 36.11% to 11 ppb.

### (c) Behavioural response of bees to rearrangement of feeder positions

We found no effect of changing the arrangement of the feeders between the first (Period 1) and second (Period 2) half of the experiment on feeding rate (LMM, *t* = 0.756, *p* > 0.451; electronic supplementary material, table S3), or on the proportion of visits to each concentration at a colony or at an individual level (GLMM, *z* < 0.158, *p* > 0.874; electronic supplementary material, tables S4 and S7). This indicates that the foraging bees detected the change in position and adjusted their behaviour to continue feeding on their preferred concentration rather than simply continuing to visit the same spatial positions they visited in Period 1.

## 4. Discussion

We demonstrate that, when presented with a choice of foraging on sucrose containing either 0, 2 or 11 ppb thiamethoxam, bumblebee (*B. t. audax)* workers increasingly prefer to visit and consume food containing thiamethoxam, thus showing the development of an acquired preference. This provides an important insight into the dynamics of foraging behaviour when bees are chronically exposed to neonicotinoid. Importantly, our initial observations of foraging visits provided no evidence of an initial preference for thiamethoxam-treated food. Moreover, when considering committed (tagged) foragers we found evidence of an initial preference for the 0 ppb sucrose over both the other two concentrations, indicating that bumblebees avoid 2 ppb and 11 ppb thiamethoxam when first applied in the environment. The development of a preference for thiamethoxam over prolonged exposure contradicts the hypothesis that bumblebees could learn to avoid a neonicotinoid-contaminated food source if alternative uncontaminated food sources are available. Due to this maladaptive behaviour, the bees increasingly retrieve more of the neonicotinoid to the colony than would be expected if the bees were foraging at random between concentrations. Our results imply that the use of thiamethoxam on flowering crops may result in the treated crops becoming disproportionately attractive to foraging bumblebees, and so further increase the risk of dietary exposure to these insecticides in wild bees [5]. For example, at the colony level, by the end of the experiment the 2 ppb concentration received 10% more foraging visits than it did at the start of the experiment. While such an increase in the uptake of the pesticide may be biologically relevant in isolation, it is worrying that we observed a consistent increase in the consumption of pesticide-treated food. This suggests that this pattern may continue if exposure persists for longer than 10 days. We also investigated the response of bees to a spatial rearrangement of the concentrations midway through the experiment. Importantly, after we changed the positions of the feeders, bees adjusted their behaviour to continue preferentially feeding from thiamethoxam-treated sucrose indicating that bumblebees possess a sensory mechanism that can detect thiamethoxam.

Our three-choice assay showed that while we detected an increasing preference for thiamethoxam, the foraging patterns between the 2 and 11 ppb concentrations were not uniform. More specifically, while the proportion of visits to the 2 ppb concentration began from a relatively low level and steadily increased throughout the experiment, the proportion of visits to the 11 ppb concentration remained similar. We also found no significant difference in feeding time per feeder visit between concentrations. Assuming feeding time predicts the volume of sucrose consumed, this dismisses the possibility that variation in the proportion of foraging visits to each concentration could have been counteracted by shorter feeding times (i.e. lower consumption per feeder visit). Dose-dependent effects on behaviour have been observed when exposing bumblebees to varying concentrations of floral toxins. For example, the presence of nicotine across a gradient of concentrations can alter the attractiveness of food to bumblebees. Baracchi *et al.* [35] showed that bumblebees are more attracted to visiting feeders containing low levels of nicotine but this effect disappeared when the concentration of nicotine was high, but within a natural range. Finally, when nicotine was provided at an artificially high concentration the proportion of visits was reduced. It was therefore plausible that synthetic chemicals, such as thiamethoxam, could stimulate similar effects on foraging. This view is supported by previous studies reporting a decrease in the consumption of neonicotinoid-laced sucrose as the concentration increased [5,16].

Our study was not designed to specifically identify the mechanism by which bees detect and develop a preference for thiamethoxam, and it is plausible that both pre- and post-ingestive mechanisms act in concert to explain the observed acquired preference. It is interesting to note that neonicotinoids excite the nicotinic acetylcholine receptors associated with learning and memory [36]. Therefore, it is possible that low concentrations of neonicotinoids act in a manner similar to low doses of naturally occurring alkaloids, like caffeine and nicotine, to provide a memorable psychoactive signal thus acting as a post-ingestive stimulant that can encourage bees to remain faithful to contaminated food sources [35,37]. Alternatively, while bumblebees are unable to detect neonicotinoids with the sensilla of their mouthparts [5], we also cannot rule out their ability to detect neonicotinoids using chemoreceptors on other parts of the body, such as the antennae or tarsi [38]. We found that changing the placement of the feeders had no effect on the rates of consumption or on the rates of change at which each concentration was visited, indicating that workers can rapidly perceive the change in their environment and correspondingly adjust their behaviour. This suggests that they can directly detect the pesticide in solution. However, to conclusively demonstrate that bumblebees can detect thiamethoxam would require testing on individual bees using a combination of behavioural assays. For example, variants of the proboscis extension response could be employed to differentiate detection through olfaction [39] or contact chemoreception by sensory sensilla not present in the proboscis [40]. The behavioural results could then be followed by confirmation of the sensory pathways using electrophysiology, similar to a previous study by Kessler *et al.* [5].

To date, many experiments revealing the effects of pesticide exposure on individual behaviour have done so by isolating one or a few workers for observation, but foraging behaviour in bumblebees is a social behaviour. Normal foraging behaviour requires a worker to leave the colony, collect food and return and deposit the food in the colony, repeating this task multiple times per day. Therefore, effects on an individual outside of this social context may not scale up to the colony level. To avoid this potential issue we used intact queenright colonies connected to a free flight arena to allow for more natural foraging behaviour. Together, our results support the conclusion that the amount of active ingredient brought back to the colony is higher than would be expected if foraging had been random, thus increasing the risk of detrimental impact on colony performance and fitness posed by neonicotinoid exposure to the colony [13–15,17,22,23,41]. Monitoring the behaviour of multiple colonies and individuals within the laboratory did, however, place a limit on the size of arenas we could use. The foraging arena we used is obviously smaller than a natural landscape. Therefore, the likelihood of interactions between foragers is increased, which could potentially influence the foraging preference we observed. For example, bumblebees in flight arenas are known to use social cues from other foragers, such as the presence of conspecifics on feeders, to help identify novel and rewarding food [42]. However, while social cues are potentially important, we argue that if such behaviour had significantly biased our results then we might expect to see a reinforcement of the initial behavioural patterns (e.g. attraction to the 0 ppb concentration), yet we observed a progressive avoidance of the 0 ppb concentration. Furthermore, even if such social learning contributed to the patterns we observed, it is important to understand why sufficient bees consistently identified the thiamethoxam-treated sucrose as a rewarding food source.

To conclude, our results imply that foraging bees may actively seek out neonicotinoid-treated flowering crops or contaminated wildflowers as the season progresses (also see [5]). If neonicotinoid-treated flowering crops or contaminated wildflowers become disproportionately attractive to foraging bees, then ironically, this could temporarily increase crop pollination but simultaneously increase dietary exposure to these insecticides [4]. This would be concerning given neonicotinoid exposure can detrimentally affect motor functions, learning, orientation and navigation, which could reduce individual foraging performance [36,43,44]. This in turn could affect colony functioning leading to reduced colony growth and the number of new reproductive individuals produced [17,22,36], particularly when in combination with other local environmental stressors such as pathogens or food stress [16,41,45,46]. Here, we highlight the need to incorporate the attractiveness of field-relevant concentrations of pesticides to foraging bees in combination with the proportion of pesticide-contaminated floral resources within the foraging range of social bees. We therefore reiterate the importance of distinguishing between the hazards posed by pesticides in the environment to bees and the risk of exposure.

## Supplementary Material

SI: Foraging bumblebees acquire a preference for neonicotinoid treated food with prolonged exposure

## Data accessibility

The data and associated code for analysis has been submitted to the Dryad repository (http://dx.doi.org/10.5061/dryad.4f6t3b5) [47].
