# Supplementary material for: Foraging bumblebees acquire a preference for neonicotinoid-treated food with prolonged exposure
Source: Proc Biol Sci. 2018 Aug 29;285(1885):20180655. doi: 10.1098/rspb.2018.0655 (PMC6125916; doi:10.1098/rspb.2018.0655)
Supplement: SI: Foraging bumblebees acquire a preference for neonicotinoid treated food with prolonged exposure [file rspb20180655supp1.pdf]

Foraging bumblebees acquire a preference for neonicotinoid treated food with prolonged exposure

Andres N. Arce[1], Ana Ramos-Rodrigues[1], Jiajun Yu[1], Thomas J. Colgan[2], Yannick Wurm[2] & Richard J. Gill[1]

Supplementary Materials

Supplementary Methods

Feeder treatment preparations

We chose a 30% sucrose solution for the experiment, which represents a similar concentration to those found in flowering crops, including oilseed rape (a.k.a. Canola; with 10-30% sugar concentration across varieties[1]). Oilseed rape is also a mass flowering crop that: i) is commonly treated with neonicotinoids across Europe (native range of *B. terrestris*); ii) neonicotinoids are frequently detected in the nectar and pollen (see Table S1); and iii) is known to be visited by many bee species[1].

The 0, 2 and 11ppb thiamethoxam solutions were prepared using a similar method for making imidacloprid solutions as explained in Gill *et al*. 2012[2]. We dissolved 100 mg of thiamethoxam in 100 ml of acetone to produce a primary stock solution (1mg/ml). Aliquots of this stock solution were added to the 30% sucrose solution to produce a 2 ppb and 11 ppb thiamethoxam solution. The 0 ppb solution was made by repeating this process but using an acetone stock solution. The primary stock solution was kept a freezer in single use aliquots and a new working solution was prepared at the start of day 6.

Bee husbandry and foraging arena

On arrival we culled the four largest colonies to 50 workers by randomly removing excess workers in order to maintain colonies at a manageable size (all colonies: median = 36.5 workers; range = 19-50; see Table S2). All workers were tagged with a unique numbered tag, and any drones present on arrival were removed (eight drones in three colonies). We transferred the brood, queen, and the workers from each colony into a separate wooden nest box (WNB; Figure 1) under red light, and left undisturbed for 48 hours with access to *ad libitum* 30% sucrose solution and provisioned with 4g of pollen. The foraging experiment was conducted in a laboratory under natural light and room temperature whilst connected to a foraging arena (dimensions = L100cm × W70cm × H50cm) by a clear Perspex tube (length = 150mm; ID = 19mm; Figure S1).

The foraging arena floor was covered with green Correx® and the roof was made from a transparent sheet of Perspex allowing the video camera to observe the feeders clearly from outside of the arena. The arena also had two sliding doors on the wooden sides which contained the bees when closed, but allowed researcher access to the inside of the arena when opened. The Perspex tube connecting the WNB to the arena had a trapdoor half-way down to control the flow of bees leaving the WNB, and so restricted access to the feeders to only the allotted 6 hour foraging period per day. The roof of the WNB was also a clear Perspex sheet, but this was covered with cardboard to mimic a dark nest and to stimulate foraging in a naturally lit foraging arena.

Training phase:

Prior to the start of the experiment, each colony was given a 4 day training period inside the arena to allow the foraging bees to learn how to access and feed from the feeders (See Figure S1 for details).

Video observations and monitoring feeding time

On watching the 180 hours of video footage it was common to see multiple foragers on the feeders simultaneously, therefore the observer scoring the behaviour was required to focus on one forager and then move on to the next by pausing and rewinding the footage. This resulted in the 180 hours of video footage being re-watched multiple times. For the analysis of feeding time we filtered the data to include only fully committed foragers to ensure that we were monitoring the behaviour of individuals that were regularly foraging on the treated sucrose, to maximise the chance of detecting a chronic effect of thiamethoxam. This retained 3851 foraging bouts conducted by 31 tagged workers (from a total of 4663 bouts by 74 tagged workers); whilst this removed half of the observed tagged workers it retained 82.5% of all the data, supporting our method of identifying committed foragers.

Supplementary analyses

Foraging visits using count data: By reporting only proportional visitation data it may not be clear what changes are driving our results i.e. it is possible that bees are either increasing their visits to food containing the pesticide, or that the visits to the 0 ppb solution are decreasing, or both. In addition to the analysis on the proportion of visits in the main text, we analysed the counts of foraging visits showing that the number of visits to each treatment increased through time (Figure S2; Table S5). The GLMM was similar to that used

to analyse the proportional data as it included the interaction between *day* and *treatment*, the effect of *period* as fixed effects, and accounted for the repeated measures by including colony as a random intercept and day as a random slope. The response variable was the number of visits and the model used a Poisson distribution. We scaled the continuous variable *day* which allowed the model to converge. Our statistical results are identical in terms of the direction of the effect and significance in everything but the effect of *period* which in this case indicates that there are more foraging visits in the second period, which we would expect as the colonies increase in size through the experiment.

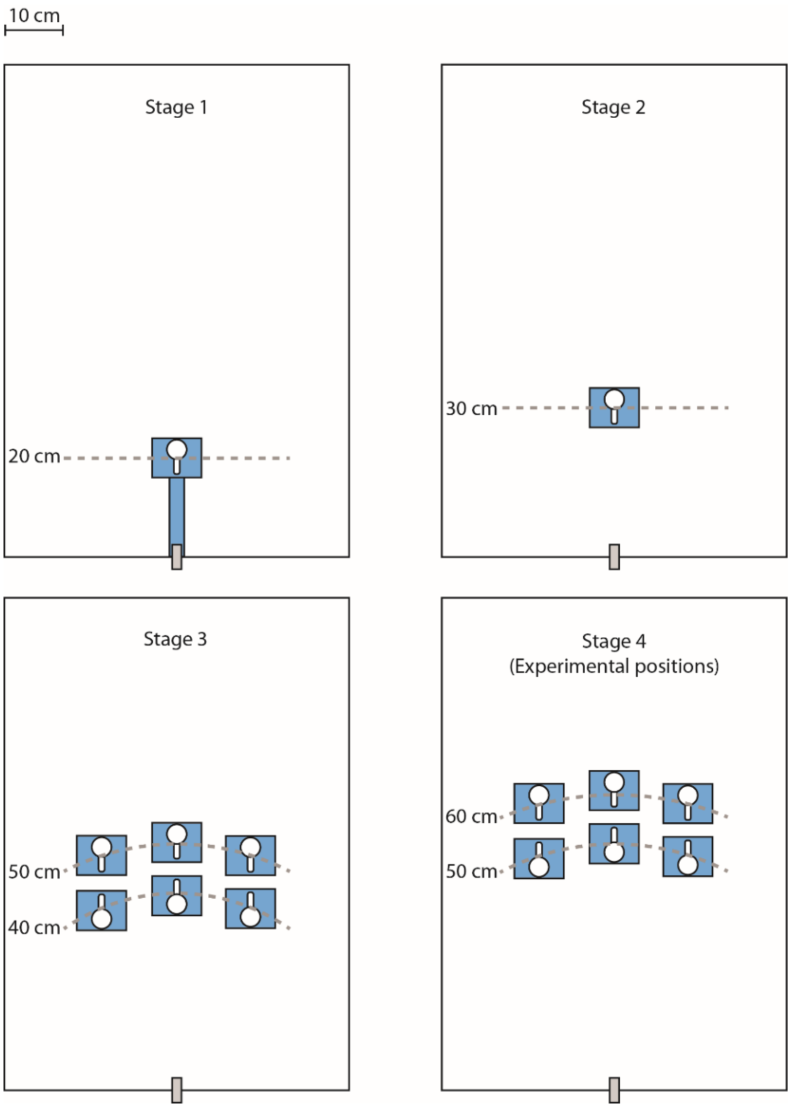

Figure S1: The training phase was conducted across 4 days. *Stage 1*: Colony connected to the foraging arena (entrance tube is indicated in grey) and a gravity feeder positioned 20 cm away from the entrance on a 7 cm high platform. The feeder was placed on top of a (10 × 8 cm) square of blue laminated card to maintain the association between the colour blue and sucrose. The platform was connected to the entrance using a bridge made from laminated blue card (3 × 20 cm), which allowed the bees to access the feeder without flying. This configuration of feeder was maintained until the workers had learnt to forage from the feeder, we defined learning as either observing at least two foragers successfully feeding, or the colony consuming at least 5 ml sucrose per day. *Stage 2*: The bridge was removed and feeder placed 30 cm from the entrance to the foraging arena. *Stage 3*: The single feeder was replaced with six identical feeders in two rows of three with each facing the other. The feeders in each row were placed 40 cm and 50 cm away from the entrance. *Stage 4*: The six feeders were moved further away from the entrance with first row being at 50 cm (close) or 60 cm (far).

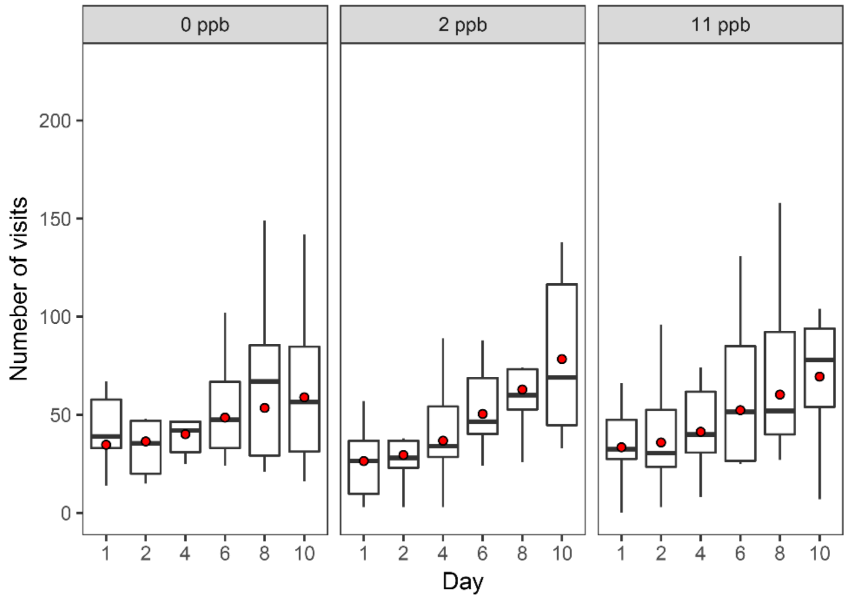

Figure S2: Boxplot showing the median, interquartile range and outliers for the number of foraging visits observed to either 0 ppb, 2 ppb, and 11 ppb thiamethoxam solutions by all workers

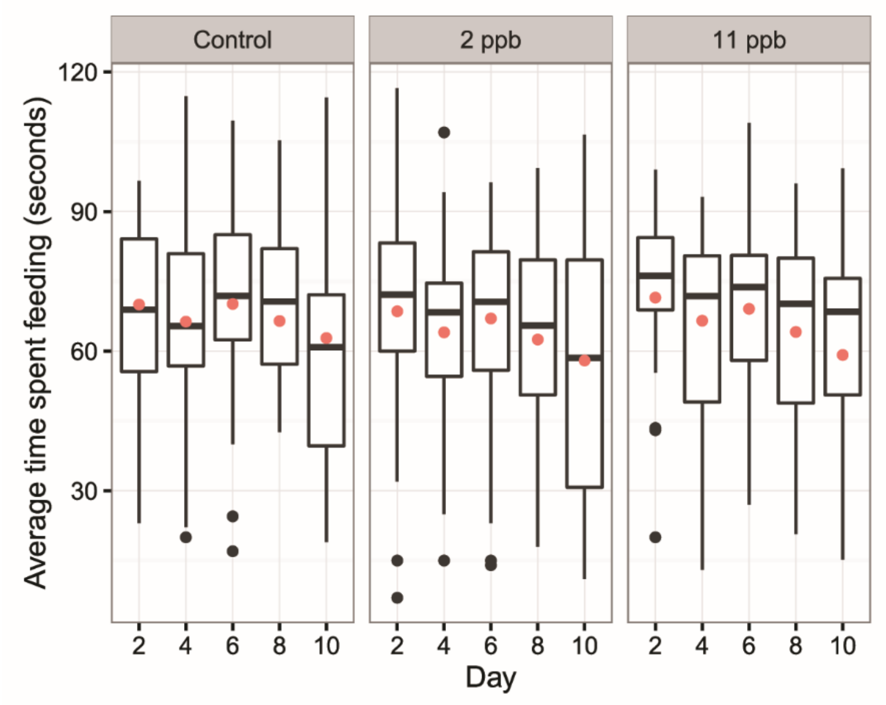

Figure S3: Boxplot showing the median, interquartile range and outliers for the time spent foraging on either 0 ppb, 2 ppb, and 11 ppb thiamethoxam solutions by tagged workers identified as being committed foragers. Red circles overlaying each box is the back transformed predictions from the mixed effects model (Table S5).

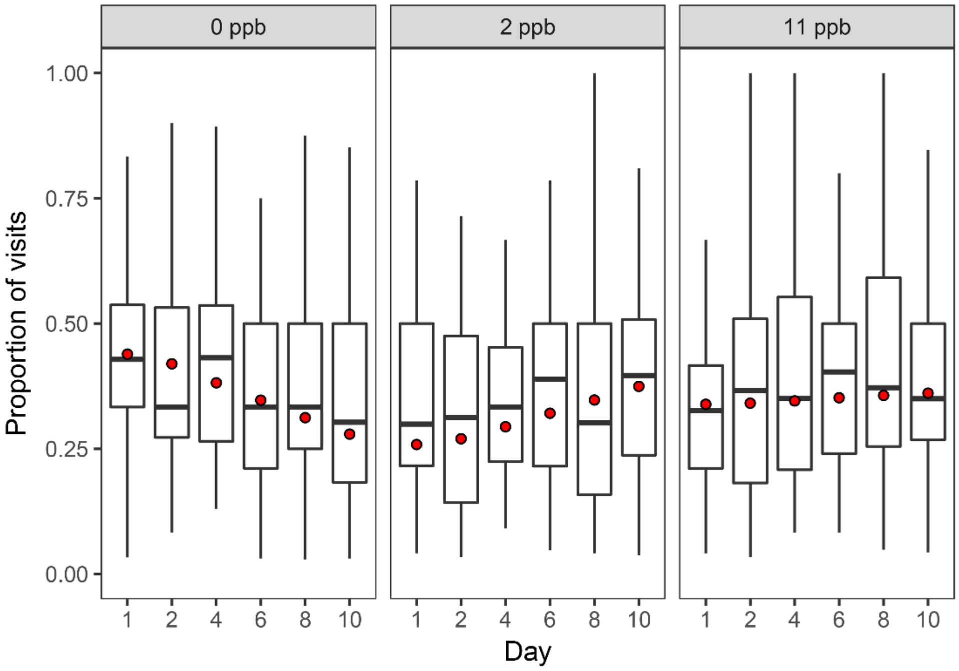

Figure S4: The proportion of foraging trips made by individuals observed foraging on at least three separate days (31 bees). Red circles represent the back transformed mean predictions from the mixed effects models.

Table S1: Selection of studies reporting the mean and ranges of thiamethoxam residues from various environmental sources. HB = honeybee, OSR=Oilseed Rape, WW = winter wheat, (-) = missing value.

**Source**		**Mean**	**Range**	**Units**	**Study**
Nectar	OSR flowers	3.2	0.1-13.3	ng/g	Botías *et al. 2015[3]*
	OSR nectar and HB honey	4.2	0-12.9	ng/g	Pohorecka *et al. 2012[4]*
	wildflowers from OSR margins	0.1	0.1-1.8	ng/g	Botías *et al. 2015[3]*
Pollen	HB	53.3	(-)	ppb	Mullin *et al. 2010*[5]
	HB during OSR bloom	0.15	0-1.6	ppb	David *et al.* 2016[6]
	OSR	5.7	2.4-11	ppb	David *et al.* 2016[6]
	OSR margin, wildflowers	2.8	0-21	ppb	David *et al.* 2016[6]
	OSR pollen and HB pollen bread	3.8	0-9.9	ng/g	Pohorecka *et al. 2012[4]*
	WW margins, wildflowers	0.13	0-0.5	ppb	David *et al.* 2016[6]
	HB during OSR bloom	0.2	0.12-1.81	ng/g	Botías *et al. 2015[3]*
	OSR flowers	3.26	1.02-11.1	ng/g	Botías *et al. 2015[3]*
	wildflowers from OSR margins	14.81	0.12-86.2	ng/g	Botías *et al. 2015[3]*
	wildflowers from WW margins	0.14	0.12-7.47	ng/g	Botías *et al. 2015[3]*
Soil	field margin	0.72	0.28-1.76	ng/g	Botías *et al. 2015[3]*
	OSR cropland	3.46	0.49-9.75	ng/g	Botías *et al. 2015[3]*
	WW field margin	0.18	0-0.45	ng/g	Botías *et al. 2015[3]*

Table S2 – Census per experimental colony: a) 'On arrival' from the commercial supplier the number of adult workers (*start*) was counted and where applicable were culled to reduce colony size to 50 workers. We also removed any *males* and we then tagged all colony *workers*; b) 'After the experiment' had ended we recorded the number of *untagged* bees in the colony, any males present (*males*), any bees that were found *dead* during the experiment, and we counted the total number of live tagged and untagged *workers*.

**Colony**	**a) On arrival**				**b) After the experiment**			
	start	*culled*	*males*	*workers*	*untagged*	*males*	*dead*	*live workers*
1	90	40	0	50	121	0	0	171
2	106	56	0	50	95	0	0	145
3	30	0	0	30	27	0	0	57
4	30	0	0	30	51	0	1	80
5	30	0	3	30	12	7	0	42
7	110	60	1	50	110	10	7	153
8	100	50	0	50	96	0	0	146
9	43	0	0	43	46	0	2	87
10	29	0	4	29	35	0	0	64
11	19	0	0	19	27	0	2	44

Table S3: LMM output for the average volume of sucrose consumed. Data was analysed using a Gaussian distribution.

	Estimate	Std. Error	df	t	p
(Intercept)	7.58556	0.57458	21.76	13.202	< 0.001
Day	0.05838	0.12543	26.34	0.465	0.645
Treatment 2ppb	-1.64667	0.54601	275	-3.016	0.003
Treatment 11ppb	-0.36	0.54601	275	-0.659	0.510
Period	0.31667	0.41915	275	0.756	0.451
Day : treatment 2ppb	0.40121	0.088	275	4.559	< 0.001
Day : treatment 11ppb	0.25364	0.088	275	2.882	0.004

Table S4: GLMM output for the proportion of bees visiting each treatment group for all observed foraging visits. Data was analysed using a Binomial distribution.

	Estimate	Std. Error	z value	Pr(>|z|)
(Intercept)	-0.49783	0.047685	-10.44	< 0.001
Day	-0.04259	0.010087	-4.223	< 0.001
Treatment 2ppb	-0.50144	0.067154	-7.467	< 0.001
Treatment 11ppb	-0.09023	0.06568	-1.374	0.169
Period	0.003348	0.054066	0.062	0.951
Day : treatment 2ppb	0.093038	0.009785	9.508	< 0.001
Day : treatment 11ppb	0.033234	0.009686	3.431	< 0.001

Table 5: GLMM output for the number of bees visiting each treatment group for all observed foraging visits. Data was analysed using a Poisson distribution.

	Estimate	Std. Error	z value	Pr(>|z|)
(Intercept)	3.499286	0.165786	21.107	< 2e-16
day	0.048218	0.010685	4.513	6.39E-06
treatment2 ppb	-0.33485	0.054953	-6.093	1.11E-09
treatment11 ppb	-0.06015	0.052723	-1.141	0.25397
periodP2	0.094692	0.0443	2.137	0.03256
day:treatment2	0.062039	0.007985	7.77	7.87E-15
day:treatment11	0.022473	0.007852	2.862	0.00421

Table S6: LMM output for the time spent feeding at each treatment group. Data was analysed using a Gaussian distribution.

	Estimate	Std. Error	df	t	p
(Intercept)	78.7533	4.1318	19.42	19.060	4.93e-14
Day	-1.9261	0.6212	77.26	-3.101	0.00269
Treatment 2ppb	-3.6739	3.8258	125	-0.96	0.33876
Treatment 11ppb	-5.7692	3.8258	125	-1.508	0.13408
Period	4.4595	2.7188	125	1.64	0.10347
Day : treatment 2ppb	0.5271	0.5768	125	0.914	0.36254
Day : treatment 11ppb	0.8592	0.5768	125	1.49	0.13884

Table S7: GLMM output for the proportion of foraging trips made by 31 individual bees to each treatment group. Data was analysed using a Binomial distribution.

	Estimate	Std. Error	z value	p
(Intercept)	-0.16621	0.069412	-2.395	0.0166
Day	-0.07893	0.01637	-4.822	1.42E-06
Treatment 2ppb	-0.94622	0.099521	-9.508	< 2e-16
Treatment 11ppb	-0.51198	0.095861	-5.341	9.25E-08
Period	0.007742	0.085227	0.091	0.9276
Day : treatment 2ppb	0.138181	0.015819	8.735	< 2e-16
Day : treatment 11ppb	0.088917	0.015506	5.734	9.79E-09

Supplementary References

1. Pierre J., Mesquida J., Marilleau R., Pham-Delègue M.H., Renard M. 1999 Nectar secretion in winter oilseed rape, Brassica napus— quantitative and qualitative variability among 71 genotypes. *Plant Breeding* **118**(6), 471-476. (doi:10.1046/j.1439-0523.1999.00421.x).
2. Gill R.J., Ramos-Rodriguez O., Raine N.E. 2012 Combined pesticide exposure severely affects individual- and colony-level traits in bees. *Nature* **491**(7422), 105-108. (doi:https://doi.org/10.1038/nature11585).
3. Botías C., David A., Horwood J., Abdul-Sada A., Nicholls E., Hill E., Goulson D. 2015 Neonicotinoid residues in wildflowers, a potential route of chronic exposure for bees. *Environmental Science & Technology* **49**(21), 12731-12740. (doi:10.1021/acs.est.5b03459).
4. Pohorecka K., Skubida P., Miszczak A., Semkiw P., Sikorski P., Zagibajło K., Teper D., Kołtowski Z., Skubida M., Zdańska D., et al. 2012 Residues of Neonicotinoid Insecticides in Bee Collected Plant Materials from Oilseed Rape Crops and their Effect on Bee Colonies. *Journal of Apicultural Science* **56**(2), 115. (doi:10.2478/v10289-012-0029-3).
5. Mullin C.A., Frazier M., Frazier J.L., Ashcraft S., Simonds R., vanEngelsdorp D., Pettis J.S. 2010 High Levels of Miticides and Agrochemicals in North American Apiaries: Implications for Honey Bee Health. *PLoS ONE* **5**(3), e9754. (doi:10.1371/journal.pone.0009754).
6. David A., Botías C., Abdul-Sada A., Nicholls E., Rotheray E.L., Hill E.M., Goulson D. 2016 Widespread contamination of wildflower and bee-collected pollen with complex mixtures of neonicotinoids and fungicides commonly applied to crops. *Environment International* **88**, 169-178. (doi:http://dx.doi.org/10.1016/j.envint.2015.12.011).
